# Form, function and phylogeny: comparative morphometrics of Lake Tanganyika's cichlid tribe Tropheini

**DOI:** 10.1111/zsc.12110

**Published:** 2015-03-10

**Authors:** Katrin A. Wanek, Christian Sturmbauer

**Affiliations:** ^1^ Department of ZoologyUniversity of GrazUniversitätsplatz 2A‐8010GrazAustria

## Abstract

Lake Tanganyika's cichlid fishes represent one of the most diverse species assemblages of the world. In this study we focused on the tribe Tropheini which occupies several trophic niches, mostly in rocky habitats. We analysed morphological variation of seventeen closely related species by means of geometric morphometric methods and related these data to ecological characteristics and phylogeny of the study species. It turned out that morphology mostly correlated well with ecological parameters, but not always closely with the degree of the phylogenetic relatedness of the species. Overall, body shapes in the tribe Tropheini are of great evolutionary plasticity, but variation is restricted to particular body parts: the preorbital region once again emerged as a key factor that facilitated their impressive radiation.

## Introduction

What are the origins of biodiversity? Adaptive radiation is one of the most impressive features of evolution and a main mechanism for the formation of finely interacting species communities. The process can be summarized as the evolution of ecological and phenotypic diversity within a rapidly multiplying lineage; a process that connects speciation with phenotypic adaption to divergent environments (Schluter 2000). Schluter employed four criteria for the detection of adaptive radiation: common ancestry, phenotype–environment correlation, trait utility and rapid speciation. The concept goes back to the 1950s (Brooks 1950; Simpson 1953), and since then many examples were described that meet the criteria: Darwin's finches (Grant & Grant 2003), Hawaiian *Drosophila*, honeycreepers and silverswords (Dominey 1984; Carlquist *et al*. 2003; Lerner *et al*. 2011), Caribbean *Anolis* lizards (Harmon *et al*. 2003; Losos *et al*. 1998) and cichlid fishes (Fryer & Iles 1972).

The family of cichlid fishes comprises more than 10% of all teleost fishes and has colonized the freshwater habitats of India, Madagascar, Africa, South and Central America (Salzburger & Meyer 2004). While their overall distribution mirrors vicariance (Chakrabarty 2004), cichlid fishes have repeatedly undergone adaptive radiation within the ecosystem of a lake by diversifying into almost every ecological niche and producing a multitude of body shapes, trophic specializations, colours and behaviours (Fryer & Iles 1972; Greenwood 1984; Sturmbauer *et al*. 2011). Their enormous success has been attributed to effective brood care and efficient adaptation of their trophic anatomy. It has recently been shown that parallel adaptive evolution into corresponding niches leads to impressive body shape similarities across lineages (Cooper *et al*. 2011; Muschick *et al*. 2012) and lakes (Kocher *et al*. 1993; Cooper *et al*. 2010; Elmer *et al*. 2010; Colombo *et al*. 2013). In fact, specific changes in trophic anatomy have evolved repeatedly in the African rift lakes via relatively simple morphological alterations (Cooper *et al*. 2010).

In this study, we address the scope of trophic adaptations within a lineage of the oldest and most mature cichlid fish species flock in Lake Tanganyika, the tribe Tropheini. By analysing 17 of the currently 25 described species with means of geometric morphometric methods, we relate the observed morphological diversity to ecological and phylogenetic patterns, by analysing 17 of the currently 25 described species (Koblmüller *et al*. 2008a; Takahashi & Koblmüller 2014). We explore the morphospace of this lineage and focus on, the head region as the main expression of trophic adaptation (Cooper *et al*. 2010, 2011; Parsons *et al*. 2011; Muschick *et al*. 2012). The aim was to reveal whether a correlation exists between morphology, phylogenetic relatedness and/or ecological specialization: Which species are most alike concerning their body shapes? Are those species that resemble each other the most, also most closely related in terms of common ancestry? Do similar ecological parameters correlate with similar body shapes?

## Background

Lake Tanganyika contains the oldest persisting adaptive radiations of several fish and invertebrate groups in the African rift region and holds a key position in the African cichlid fish fauna, both as evolutionary reservoir of ancient and birth place of novel cichlid lineages (Salzburger *et al*. 2002, 2005). The Tanganyika radiation was seeded by at least seven ancient riverine lineages present in the Proto‐Malagarazi‐Congo River, which diversified in parallel (Salzburger *et al*. 2002): the ancestors of the substrate breeding predator *Boulengerochromis microlepis*, the genus *Hemibates* and of the Bathybatini, Trematocarini, Eretmodini, the non‐mouthbrooding Lamprologini, as well as of the C lineage (*sensu* Clabaut *et al*. 2005). Some of the emerging lineages actually left the lake area to colonize other rivers and lakes: the lamprologines (Sturmbauer & Meyer 1993; Sturmbauer *et al*. 2010) and the haplochromines (Salzburger *et al*. 2005; Koblmüller *et al*. 2008a).

The mouthbrooder radiation within the C lineage particularly highlights the lake–river interphase, as it contains both riverine and lacustrine clades. It was suggested to have diversified rapidly after the change of the lake ecosystem from a shallow more swamp‐like lake habitat to a truly lacustrine habitat (Sturmbauer 1998; Salzburger *et al*. 2002). The haplochromines spread over almost all major river systems of Africa containing a set of non‐eggspot‐carrying ancestral lineages plus the egg‐spot‐carrying modern haplochromines. The modern haplochromines consist of the Tanganyika‐endemic Tropheini on the one hand and a complicated array of riverine and lacustrine haplochromines including the flocks of Kivu, Malawi, Victoria and Turkana, on the other (Verheyen *et al*. 2003; Salzburger *et al*. 2005; Koblmüller *et al*. 2008b). As part of the Tanganyika radiation, the ancestors of the haplochromines evolved additional key innovations (i.e. maternal mouthbrooding, sexually selected egg spots and colour polymorphism) to enable them to undergo replicate adaptive radiations whenever ecological opportunities arose through the formation of a (larger) lake (Wagner *et al*. 2012). The tribe Tropheini (Poll 1986) stands out for several reasons: it diversified within the confines of the lake after the initial diversification and spread of the non‐endemic ancestral haplochromines. At the same time, it is the sister group and more ancient ecological equivalent of all other lacustrine modern haplochromines that diversified later and independently to endemic species assemblages in Lakes Malawi, Kivu, Victoria, Albert and Turkana (Verheyen *et al*. 2003; Koblmüller *et al*. 2008b). The age of the Tropheini exceeds that of the entire Lake Malawi cichlid species flock, and its roots can be traced back to species living also in the rivers and swamps connected to Lake Tanganyika, from which they split about 2.8 mya (Koblmüller *et al*. 2010). However, all extant tribe members are lake endemics so that they have radiated into several available niches of the littoral zone 1.5–2.1 mya, mostly in rock and cobble habitats, probably from generalist swamp‐dwelling modern haplochromine ancestors. Three of the endemic species, *C. horei*,* S. diagramma* and *S. babaulti*, also enter swamps and rivers (Kullander & Roberts 2011) where the entire tribe is presumed to have originated. In terms of eco‐morphological diversity, the Tropheini speciated into an impressive range of trophic niches, ranging from small fish and invertebrate predators, snail eaters, omnivores to various types of epilithic algae and detritus feeders, also termed aufwuchs eaters (Yamaoka 1997; Sturmbauer *et al*. 2003; Koblmüller *et al*. 2010). The specific adaptation to particular ecological niches often led to a reduction of the ability for dispersal over ecological barriers which resulted in numerous distinct populations, geographical races, colour morphs and sister species (Sturmbauer & Meyer 1992; Sturmbauer *et al*. 2003, 2005; Egger *et al*. 2007; Wagner & McCune 2009; Koblmüller *et al*. 2011; Van Steenberge 2014). It follows that each shore line is inhabited by a unique set of tropheini entities, comprising both more widespread species and local endemics, depending on the dispersal capacity and history of each species.

## Material and methods

### Sampling and data acquisition

We analysed 396 specimens belonging to seventeen species of the tribe Tropheini listed in Table 1. To place the focus on truly interacting members of a species community, but to also cover the diversity of the tribe, we took the following sampling approach: wherever possible we used species from a single cobble shore at the south‐eastern edge of the lake south of Kalambo River estuary (coordinates: S 8°37′, E 31°12′), resulting in a sample of 13 truly sympatric species. To cover all trophic types, we additionally included samples of previously caught adult fish that were kept alive at the fish facility of Toby Veall (*Petrochromis trewavasae*,* Petrochromis ephippium*,* Tropheus polli* and *Tropheus duboisi*). Adult fish were captured by gill nets and put in concrete ponds with lake water. Each specimen was anaesthetized via clove oil before being measured (standard length according to Barel *et al*. 1977), sexed and scanned with a modified flatbed scanner (see Herler *et al*. 2007). The majority of the sampled individuals were released back to the lake after recovering from anesthetization. Ecological data was recovered from relevant publications (Yamaoka 1997; Konings 1999, 2005, 2013; Herrmann 2002; Froese & Pauly 2011; Muschick *et al*. 2012; Colombo *et al*. 2013) as summarized in Table 1. We focused on feeding preference as our main ecological criterion, because nutrition is tightly associated with habitat choice but can be more easily differentiated. Albeit fish tend to feed opportunistically depending on what is abundant, we grouped feeding preference in four guilds: Carnivores, omnivores and aufwuchs feeders (subdivided into algae grazers and algae browsers). *Gnathochromis pfefferi*,* Ctenochromis horei* and the snail eater *Lobochilotes labiatus* made up the carnivorous group*. Limnotilapia dardennii* is an aufwuchs feeder with a particularly high proportion of animal food and therefore was classified as omnivorous. Among the aufwuchs feeders, algae grazers were defined as those that pickup large quantities of substrate by scraping or rasping at an algae‐covered surface while algae browsers are more selective and seldom ingest substrate (Yamaoka 1991; Coleman 2000). The algae grazer group comprised all six *Petrochromis* species plus *Interochromis loocki*. The algae browser group consisted of *Pseudosimochromis curvifrons, Simochromis babaulti, Simochromis diagramma, Tropheus duboisi, Tropheus moorii* and *T. polli*. Concerning phylogenetic data of the Tropheini, we mainly refer to a recent publication by Koblmüller *et al*. (2010) in which the most inclusive evolutionary hypothesis based upon AFLP markers was presented (see also Sturmbauer *et al*. 2003; Colombo *et al*. 2013). The study used nuclear markers (AFLP) and mitochondrial sequences (the complete ND2 gene and the complete control region) and created a highly resolved phylogeny of the Tropheini. The original AFLP tree of Koblmüller *et al*. (2010) with the species included in this study highlighted, as well as a simplified tree with the species used in our study are available in the supporting information section (Figs S1 and S2). The AFLP phylogeny suggested four early and rapidly diversifying lineages, with lineage 1 consisting of all algae grazers (all *Petrochromis* species plus *I. loocki*), lineage 2 including a mixture of ecotypes (*S. diagramma*,* L. dardennii*,* G. pfefferi*,* C. horei*,* P. curvifrons* and *S. babaulti*), *lineage* 3 comprising the carnivorous *L. labiatus* and lineage 4 containing the genus *Tropheus* (*T. moorii*,* T. polli*, and at its base also *T. duboisi*).

**Table 1:** Sample list including ecological characteristics of the analysed specimens and their lineage assignment according to the AFLP phylogeny (Koblmüller *et al*. 2010)

Species	*n*	Date of sample	Sampling locality	Habitat	Feeding preference
*Ctenochromis horei* (Günther, 1893) Lineage 2	25	2010, 2011	Kalambo Lodge	Shallow, stony‐sandy or muddy, sediment‐rich habitat	Carnivorous
*Gnathochromis pfefferi* (Boulenger, 1898) Lineage 2	25	2010, 2011	Kalambo Lodge	Shallow, stony‐sandy or muddy, sediment‐rich habitat	Carnivorous
*Interochromis loocki* (Poll, 1949) Lineage 1	25	2011	Kalambo Lodge	Intermediate zone (rocky to sandy)	Aufwuchs (algae grazer)
*Limnotilapia dardennii* (Boulenger, 1899) Lineage 2	25	2011	Kalambo Lodge	Intermediate zone (rocky to sandy)	Omnivorous
*Lobochilotes labiatus* (Boulenger, 1898) Lineage 3	25	2010, 2011	Kalambo Lodge	Intermediate zone (rocky to sandy)	Carnivorous
*Petrochromis ephippium* (Brichard, 1989) Lineage 1	25	2011	Tanzania	Rocky shores	Aufwuchs (algae grazer)
*Petrochromis famula* (Matthes & Trewavas, 1960) Lineage 1	25	2011	Kalambo Lodge	Rocky shores	Aufwuchs (algae grazer)
*Petrochromis fasciolatus* (Boulenger, 1914) Lineage 1	25	2011	Kalambo Lodge	Rocky shores	Aufwuchs (algae grazer)
*Petrochromis macrognathus* (Yamaoka, 1983) Lineage 1	3	2011	Kalambo Lodge	Rocky shores	Aufwuchs (algae grazer)
*Petrochromis polyodon* (Boulenger, 1899) Lineage 1	25	2011	Kalambo Lodge	Rocky shores	Aufwuchs (algae grazer)
*Petrochromis trewavasae* (Poll, 1948) Lineage 1	25	2011	Katete (TZ)	Rocky shores	Aufwuchs (algae grazer)
*Pseudosimochromis curvifrons* (Poll, 1942) Lineage 2	25	2011	Kalambo Lodge	Rubble areas in shallow water	Aufwuchs (algae browser)
*Simochromis babaulti* (Pellegrin, 1927) Lineage 2	25	2010, 2011	Kalambo Lodge	Shallow, stony‐sandy, sediment‐rich habitat	Aufwuchs (algae browser)
*Simochromis diagramma* (Günther, 1894) Lineage 2	25	2010, 2011	Kalambo Lodge	Shallow, stony‐sandy, sediment‐rich habitat	Aufwuchs (algae browser)
*Tropheus duboisi* (Marlier, 1959) Lineage 4	21	2006	Halembe (TZ)	Rocky shores, 3–10 m depth	Aufwuchs (algae browser)
*Tropheus moorii* (Boulenger, 1898) Lineage 4	25	2005	Mbita Island	Rocky shores, ≥1 m depth	Aufwuchs (algae browser)
*Tropheus polli* (Axelrod, 1977) Lineage 4	25	2009	Mahale (TZ)	Rocky shores, ≥1 m depth	Aufwuchs (algae browser)

### Data editing for geometric morphometric analyses

**Figure 1:**
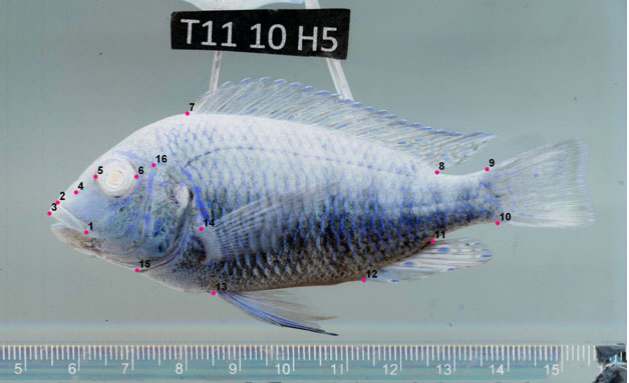
Landmarks exemplarily shown on a specimen of *Interochromis loocki*. Number and position of landmarks: 1 – corner of the mouth, 2 – topmost point of the upper lip at the transition to the nasal region, 3 – most anterior and most ventral point of the upper lip, 4 – nostril, 5 – anterior extreme of the orbit along the anterioposterior body axis, 6 – posterior extreme of the orbit along the anterioposterior body axis (a connecting line between 5 and 6 would cross the centre of the orbit), 7 – anterior origin of the dorsal fin, 8 – posterior insertion of the dorsal fin, 9 – upper origin of the caudal fin, 10 – lower insertion of the caudal fin, 11 – posterior insertion of the anal fin, 12 – anterior origin of the anal fin, 13 – origin of the ventral fin, 14 – upper origin of the pectoral fin, 15 – ventral tip of cleithrum, 16 – dorsal end of the preopercular groove.

We processed all 396 digital images with the software tpsUtil and tpsDig2 (Rohlf 2009, 2010) to obtain a landmark based data set. After comparison with corresponding studies (Kassam *et al*. 2003; Klingenberg *et al*. 2003; Chakrabarty 2005; Clabaut *et al*. 2007; Maderbacher *et al*. 2008; Herler *et al*. 2010; Kerschbaumer *et al*. 2011; Muschick *et al*. 2012) and in regard to the specific demands of our data, a landmark set with sixteen homologous points was established. We defined landmarks that were equally well detectable on all of the seventeen study species. As preliminary data and the studies aforementioned had shown that most of the interspecific differences were located in the head area, we especially focused on placing a reliable landmark block there: eight of sixteen landmarks comprised the cranial region. Taking into regard that mouth position and lip thickness are important characteristics distinguishing species and feeding types, we set three landmarks there. A second block of landmarks placed at the fin origins and insertions covered mid body and caudal region. The complete set of landmarks is shown in Fig. 1.

### Strategy of data analysis

To gain an overview of the heterogeneity of body size across the samples, we calculated arithmetic mean, standard deviation and coefficient of variation of standard length and tested for size‐dependent shape variation in our data set. As basis of all subsequent analysis, we first calculated Procrustes coordinates (see e.g. Mitteroecker & Gunz 2009) and removed size‐dependent variation using. IMP Standard7 (Sheets 2001). We then applied two ordination methods, principle component analysis (PCA) and canonical variate analysis (CVA). Principle component analysis (PCA), which is a tool for simplifying description of variation among individuals and canonical variate analysis (CVA), which simplifies description of differences between pre‐defined groups (Zelditch *et al*. 2004). We used two software packages in parallel, PAST and the IMP7 bundle (Hammer *et al*. 2001; Sheets 2001; Rohlf 2009, 2010), using PCA and CVA plots from PAST, as well as deformation grids and vector graphs from IMP. We performed a PCA of all specimens and visualized shape differences between individuals with deformation grids and vector graphics. Further, we carried out CVA of all specimens with three different grouping variables: species assignment, feeding preference and phylogenetic lineage. We tested for statistical differences between groups using MANOVA. Again, patterns of shape change were visualized with deformation grids and vectors.

## Results

### Size heterogeneity

Calculations of coefficients of variation revealed that specimens of *L. dardennii* had the highest coefficient of variation (37.3%) whereas those of *T. moorii* held the smallest (3.3%). The average coefficient of variation for all species sampled was 13% (see supporting information for details, Table S1). Knowing that sample size of *P. macrognathus* was too small to carry out meaningful statistics, we nonetheless included the three specimens to complete our array of species.

### Geometric morphometric analyses: shape and species assignment

**Figure 2:**
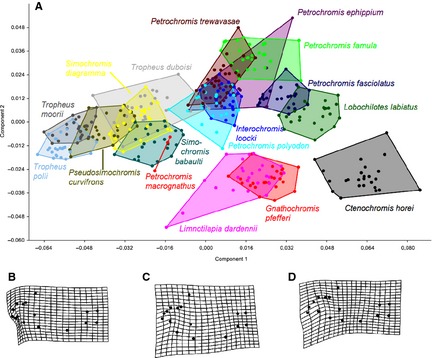
—A. PCA of all 396 specimens.—B. Pattern of shape change along PC1. —C. Pattern of shape change along PC2. —D. Pattern of shape change along PC3.

Procrustes coordinates (see supporting information, Fig. S3) already demonstrated that dispersion of individual landmarks increased around those landmarks representing the mouth region. Consequently, most of the intra‐ and interspecific differences were localized there, resulting from distinct mouth forms and positions according to different feeding habits. Least dispersion was found around those landmarks representing the caudal region. PCA resulted in three principal components with distinct eigenvalues that explained 75.3% of the variance: PC 1 explained 48.4% of variance, PC 2 15.8% and PC3 11.5% of variance. Plotting of the first two principle components (Fig. 2A) and of component 1 versus component 3 (see supporting information, Fig. S4) revealed similar clusters: *Tropheus* spp. with *Simochromis* and *Pseudosimochromis*;* Petrochromis* spp. overlapping with *Interochromis*. Shape space positions of the carnivore and omnivore species *Ctenochromis, Lobochilotes, Gnathochromis* and *Limnotilapia* varied. PCA showed that shape differences among species and genera of the Tropheini were predominately affiliated with mouth position, mouth size and body depth: Principal component 1 reflected a more inferior mouth position. Principal component 2 was associated with a deepening of the mid body (see Fig. 2B,C). Principal component 3 implicated an increase in mouth size in general and upper lip size especially as well as a slight compression of the lower mid body (Fig. 2D).

**Figure 3:**
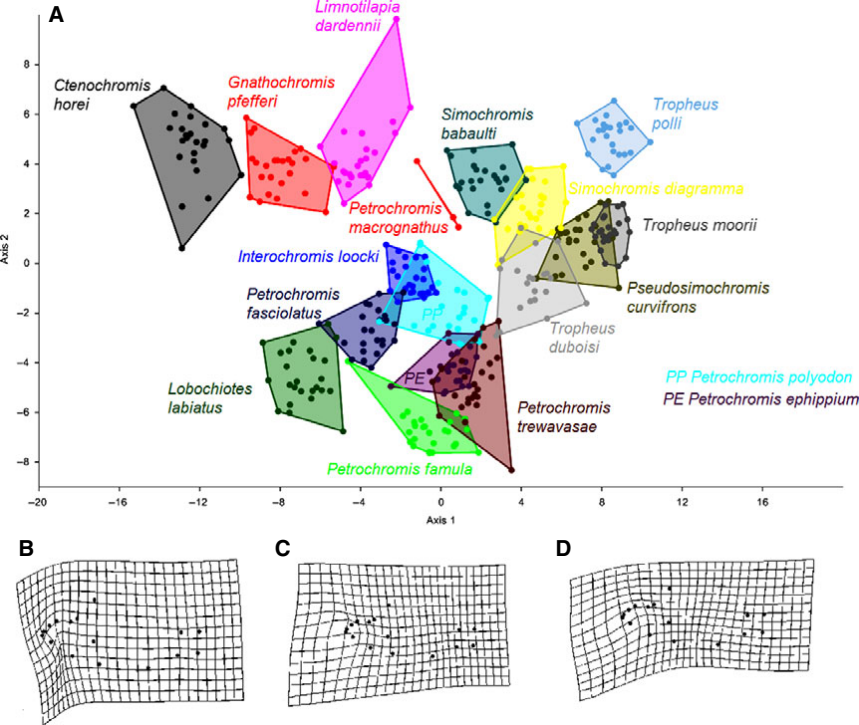
A. CVA of all 17 species. —B. Pattern of shape change along CV axis 1. —C. Pattern of shape change along CV axis 2. —D. Pattern of shape change along CV axis 3.

To address differences between species groups we continued with CVA and MANOVA. Not all species groups could clearly be separated along the two most significant CV axes (Fig. 3), as relative position of species partly overlapped. *T. moorii* and *T. duboisi* with *P. curvifrons,* and *S. diagramma*. All *Petrochromis*/*Interochromis* species clustered. Only two species occupied a fully distinct section of shape space: *C. horei* and *T. polli*. All other species partly overlapped with up to four other species. MANOVA nonetheless showed that all groups differ significantly from another. Shape changes along the first CV axis reflected a change in mouth direction towards a more ventral and inferior position along with a shorter head and a steeper forehead. CV axis 2 implied a deepening of the mid body and an increase in mouth size and upper lip size (see Fig. 3B,C). Axis 3 accounted for a change of mouth direction to a more superior position along with a deepening of the mid body (see Fig. 3D). MANOVA results are summarized in the supporting information section (Table S2), Jack‐knife test of the effectiveness of the assignment yielded 96.8% of correct and significant assignments. There were 16 distinct canonical variates with *P*‐values smaller than 0.05 and three axis with distinct eigenvalues. Axis 1 represented 37.3% of the variation, Axis 2 represented 14.6% of variation, Axis 3 represented 11.0% of variation. All other eigenvalues ranged under 5%.

### Geometric morphometric analyses: shape and ecology

**Figure 4:**
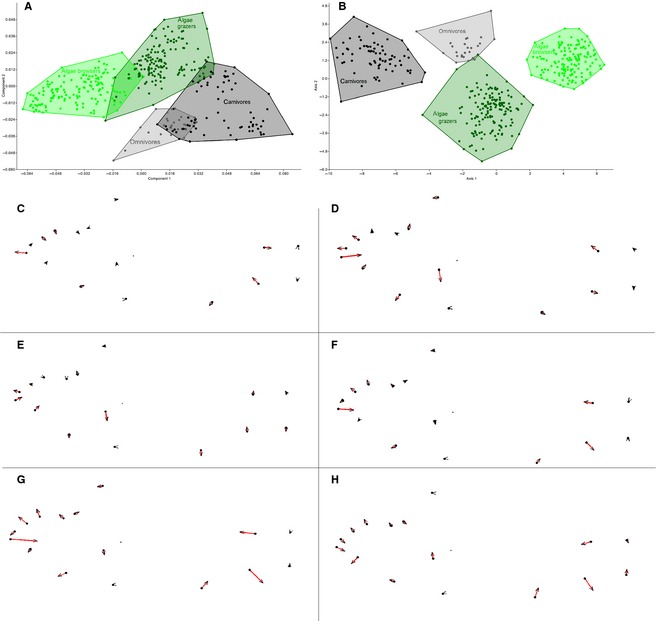
PCA (A) and CVA (B) of all 396 specimens grouped to carnivores, algae grazers, algae browsers and omnivores. Shape changes between omnivores and carnivores (C), carnivores and algae grazers (D), omnivores and algae grazers (E), omnivores and algae browsers (F), carnivores and algae browsers (G) and algae grazers and algae browsers (H), visualized through thin‐plate‐spline plots.

We subsequently tested for a correlation between body shape and trophic specialization performing a CVA and comparing results to the preceding PCA results. Results from CVA/MANOVA (see supporting information, Table S2) showed three distinct canonical variates, supported by a Jack‐knife test of the CVA performance yielding 98.5% of correct and significant assignments. Thus, trophic guilds do clearly differ in their relative position in shape space, even though their PCA shape space positioning partly overlapped (Fig. 4). *Lobochilotes labiatus* and *P. fasciolatus* were those species responsible for similarities between carnivores and algae grazers, whereas *L. dardennii* and *G. pfefferi* made shape spaces of omnivores and carnivores slightly overlap. Concerning shape differences between the four trophic groups, it turned out that the differences between omnivores and carnivores were smaller than those between algae grazers and algae browsers. Moreover, differences in shape between carnivores and aufwuchs feeders were bigger than those between omnivores and aufwuchs feeders (see Fig. 4C–H). All shape changes mainly concerned mouth position and shape.

### Geometric morphometric analyses: shape and phylogeny

**Figure 5:**
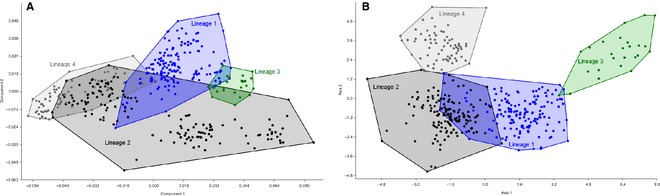
PCA (A) and CVA (B) of all 396 specimens grouped to lineages.

Results from CVA and MANOVA (Fig. 5 and supporting information, Table S2) showed significant differences between lineages, supported by a Jack‐knife test of the CVA performance yielding 95.0% of correct and significant assignments. Figure 5 gives a comparison to the preceding PCA and visualizes the clear separation of the lineages by CVA. Lineage 2 with *S. diagramma*,* L. dardenni*,* G. pfefferi*,* C. horei*,* P. curvifrons* and *S. babaulti* occupied the broadest relative shape space, according to the complex and deeply branching substructure of this clade. Although they could be clearly separated from all other groups via CVA, part of the species did overlap with *T. duboisi* from the more distantly related *Tropheus* lineage, indicating that *T. duboisi* is morphologically closer to *Simochromis*/*Pseudosimochromis* than to the other *Tropheus* species.

## Discussion

### Shape clusters and allometry

The species of the Tropheini show great variation in adult size. Therefore, removing size‐dependent variation ensured that allometry did not obscure distinctition in morphospace. We found that the in part considerable wide range in individual body size within the sampled adult individuals of certain taxa did not obscure the results of the geometric morphometric analysis: all entities emerged as clusters despite a relative wide size range and sexual dimorphism among the species. This observation is in line with previous studies on *Tropheus* (Herler *et al*. 2010; Kerschbaumer *et al*. 2011; Kerschbaumer *et al*., 2013). PCA also showed that the section of shape space occupied by each species did not correlate with its size heterogeneity: For example, *T. duboisi* and *P. ephippium*, two species with a low or average value in heterogeneity (6.8 and 14.5%), occupied relative shape spaces even larger than those of the species with the highest heterogeneity value (*L. dardennii*, 37.3%). To conclude, even if we found considerable variation in morphology among the (in most cases about 20) individuals of each taxon, they all occupied a consistent range in morphospace, albeit with some overlap with their closest morphological allies.

### Shape, species assignment and ecology

Geometric morphometric analyses of shape in relation to species assignments clearly demonstrated that the differences in shape between the 396 investigated individuals could be attributed to three main characteristics: mouth position, mouth size and body depth. The most important component accounting for 46.8% of total variance was mouth position. Differences between species could also be related to three variates, of which the most important one – causing 44.6% of interspecific differences – reflected a change in mouth direction towards a more ventral and inferior position along with a shorter head and a steeper forehead. The other two variates with significant values consisted of combinations of the characteristics already known from interindividual differences: CV axis 2 implied a deepening of the mid body and an increase in mouth size and upper lip size. Axis 3 accounted for a change of mouth direction to a more superior position along with a deepening of the mid body. These observations are in line with previous findings on the functional integration of the skull in Malawi cichlids (Cooper *et al*. 2011) and the relative independence of the anatomy of the preorbital region in relation to other head regions, demonstrated for the radiations of lakes Victoria, Malawi and Tanganyika (Parsons *et al*. 2011). This study also showed that the modularity of the preorbital region followed different trajectories in Lake Malawi ‘suction feeding’ carnivore sand‐dwellers and ‘biting’ aufwuchs‐eating rock‐dwellers consistent with ecological character displacement. The Tropheini contain carnivorous suction feeders and aufwuchs‐eating biters (we subdivided them into grazers and browsers according to Yamaoka 1991 and Coleman 2000). Our analysis shows that this division is also apparent in shape space, again consistent with differential evolutionary trajectories suggested for various African cichlids by Parsons *et al*. (2011), and ecological character displacement. A recent study on *Tropheus* populations, also indicated ecological character displacement via competitive interactions and niche segregation (Kerschbaumer *et al*. 2013).

Although MANOVA showed that species clearly differed from another, grouping results of PCA could not be directly associated with ecological or phylogenetic factors. Clusters between closely related species occurred as well as clusters between trophic guilds, which made it necessary to test for each factor separately. The Tropheini rapidly diversified via adaptation to several ecological niches and thereby specialized to specific diets in specific habitats. Adaptation towards aufwuchs feeding seems to be the most successful strategy as the majority of the species (13 of the 17 study species) have evolved this feeding strategy. The existence of only one omnivorous and three carnivorous species suggests that there are more limitations or competition for coexisting omnivores and carnivores. Also, most of the sympatric lamprologines are carnivores. The non‐monophyletic placement of algae browsers indicates that eco‐morphological specialisations related to aufwuchs feeding seem to have evolved independently and sometimes, even repeatedly (Koblmüller *et al*. 2010). The fact that shape differences between algae grazers and algae browsers were larger than those between omnivores and carnivores is in line with their evolutionary history. A recent study on sympatric algae browsers and grazers analysed stomach content in relation to total biodiversity in the epiphyton of their habitat using a pyrosequencing approach. The authors showed substantial diet disparity in the same ecomorph, caused by depth dependent shifts in the composition of biocover and foraging selectivity (Hata *et al*. 2014). This is the most inclusive account for diet spectra in algivorous species, confirming our morphology‐based separation. Yet another interesting outcome of our study was that the carnivorous (snail eater) *L. labiatus* in terms of body shape was close to algae grazers of the *Petrochromis* group: Despite their contrasting feeding preferences and dentition, they share characteristics associated with mouth form and position. It is interesting to note that Koblmüller *et al*. (2010) found traces of ancient gene flow from the *Petrochromis*/*Interochromis* ancestor into *L. labiatus*. Consisting of three species, only the carnivores group showed broad variation in body shapes due to the very distinct mouth forms of *C*. *horei*,* G. pfefferi* and *L. labiatus* (see supporting information, Fig. S5). Those large shape differences between various types of carnivores may be explained by the fact that carnivority covers a very broad range of feeding resources from small prey such as invertebrates or fry, snails, sponges to large fish. The intermediate position of the omnivore *L. dardennii* also highlights the great range of possible morphospaces for different carnivores.

### Shape and phylogeny

We are aware that – given the almost instantaneous cladogenesis at the base of the Tropheini, followed by further rapid diversification – lineage assignment is difficult, especially for lineage 2. However, CVA and MANOVA (see supporting information) showed significant differences between the four lineages defined according to Koblmüller *et al*. (2010), supported by a Jack‐knife test of the CVA performance yielding 97.2% of correct and significant assignments. Figure 5 shows our comparison of the AFLP‐based lineages to the PCA‐based upon our geometric morphometric data and visualizes partial congruence of morphology and phylogeny. Although species could be clearly separated from all other groups via CVA, the overlap of *Simochromis*/*Pseudosimochromis* with *T. duboisi* from the more distantly related *Tropheus* lineage is interesting. In terms of morphology, T. duboisi is closer to the Simochromis/Pseudosimochromis group than to all other Tropheus. The basal position of T. duboisi in the Tropheus clade reflects that fact.

Our attempt to interpret similarity in shape in the context of phylogeny indicates that only the algae grazers form an ecologically, morphologically and phylogenetically homogenous group: the *Petrochromis/Interochromis* clade. This is especially interesting as mouth shape, dentition and trophic specialization are among the most extreme in the entire cichlid family. These species have several rows of comb‐like teeth to ingest unicellular algae, microscopic invertebrates, detritus (loose biocover) from solid rock (Sturmbauer *et al*. 1992; Yamaoka 1997). They have large territories to satisfy their nutritive needs. It is interesting that the most ancestral splits (*P. fasciolatus*,* P. orthognathus* and *I. loocki*) show the least distinctive mouth enlargement and dentition, (Koblmüller *et al*. 2010). This is in line with the notion expressed by Humphrey Greenwood (1984) that ecomorphological specialization is a progressive process starting with moderate morphological divergence and beeing pushed to more and more extreme morphologies, the more time the a species flock has to evolve. This pattern was suggested for the three species flocks/assemblages of Lakes Victoria, Malawi and Tanganyika (Sturmbauer 1998), and explicitly tested by Cooper *et al*. (2010).

Algae browsers can be found in two lineages. *Tropheus duboisi*, placed as the most ancestral split of the *Tropheus* lineage (albeit with weak support), is overlapping in the morphospace with *Simochromis*/*Pseudosimochromis* and can be seen as morphological connection of the algae browsers. This also makes sense, as mouth and dentition of *T. duboisi* resemble *Simochromis*/*Pseudosimochromis* more closely than the remaining *Tropheus* species (Van Steenberge 2014). The carnivorous species and sole snail eater *L. labiatus* forms a separate lineage. In morphospace it is intermediate showing affinities with the other two carnivorous species and the omnivore *L. dardennii* as well as with the algae grazer *P. fasciolatus*. Given the particular proximity in morphospace, it seems plausible that the two carnivores *L. labiatus* and *C. horei* share similarities in body form despite separate evolutionary trajectories. The members of lineage 2 (*S. diagramma*,* L. dardennii*,* G. pfefferi*,* C. horei*,* P. curvifrons* and *S. babaulti*) displayed a broad dispersal in the morphospace. Some of their morphological affinities are congruent with the deeply branching substructuring of this clade.

To conclude, body shapes in the tribe Tropheini are of great plasticity, but variation is restricted to particular body parts: again, the cichlid feeding apparatus turns out as a modular evolutionary key factor facilitating their massive radiation. The impressive scope of morphological diversity within species of the tribe Tropheini turns out to be the result of a progressive morphological divergence in the course of closely following speciation events (Sturmbauer *et al*. 2003), which might have been triggered by lake level fluctuations (Rossiter 1995; Sturmbauer 1998; Sturmbauer *et al*. 2003). In addition to expected similarities in body shape between closely related species, we also found affinities between more distant taxa. Therefore, sharing of the same ecological traits (feeding preference in our case) often implies congruence in morphological traits, and not necessarily common ancestry, not even within a single monophyletic assemblage.

## Supporting information


**Fig. S1.** AFLP‐Phylogeny of the Tropheini after Koblmüller *et al*. 2010, p. 322.

**Fig. S2.** Phylogeny of the Tropheini, schematically after Koblmüller *et al*. (2010).

**Fig. S3.** Procrustes coordinates of all sampled specimens.

**Fig. S4. A:** PC1 of all species: PC 1 plotted against PC3.

**Fig. S5.** Heads of *Ctenochromis horei* (left), *Gnathochromis pfefferi* (center) and *Lobochilotes labiatus* (right).

**Table S1.** Arithmetic mean of standard lengths, standard deviation and coefficient of variation for all examined species.

**Table S2.** Results from CVA/MANOVA.
