# Supplementary figures and images for: Form, function and phylogeny: comparative morphometrics of Lake Tanganyika's cichlid tribe Tropheini
Source: Zool Scr. 2015 Mar 10;44(4):362–73. doi: 10.1111/zsc.12110 (PMC4949720; doi:10.1111/zsc.12110)

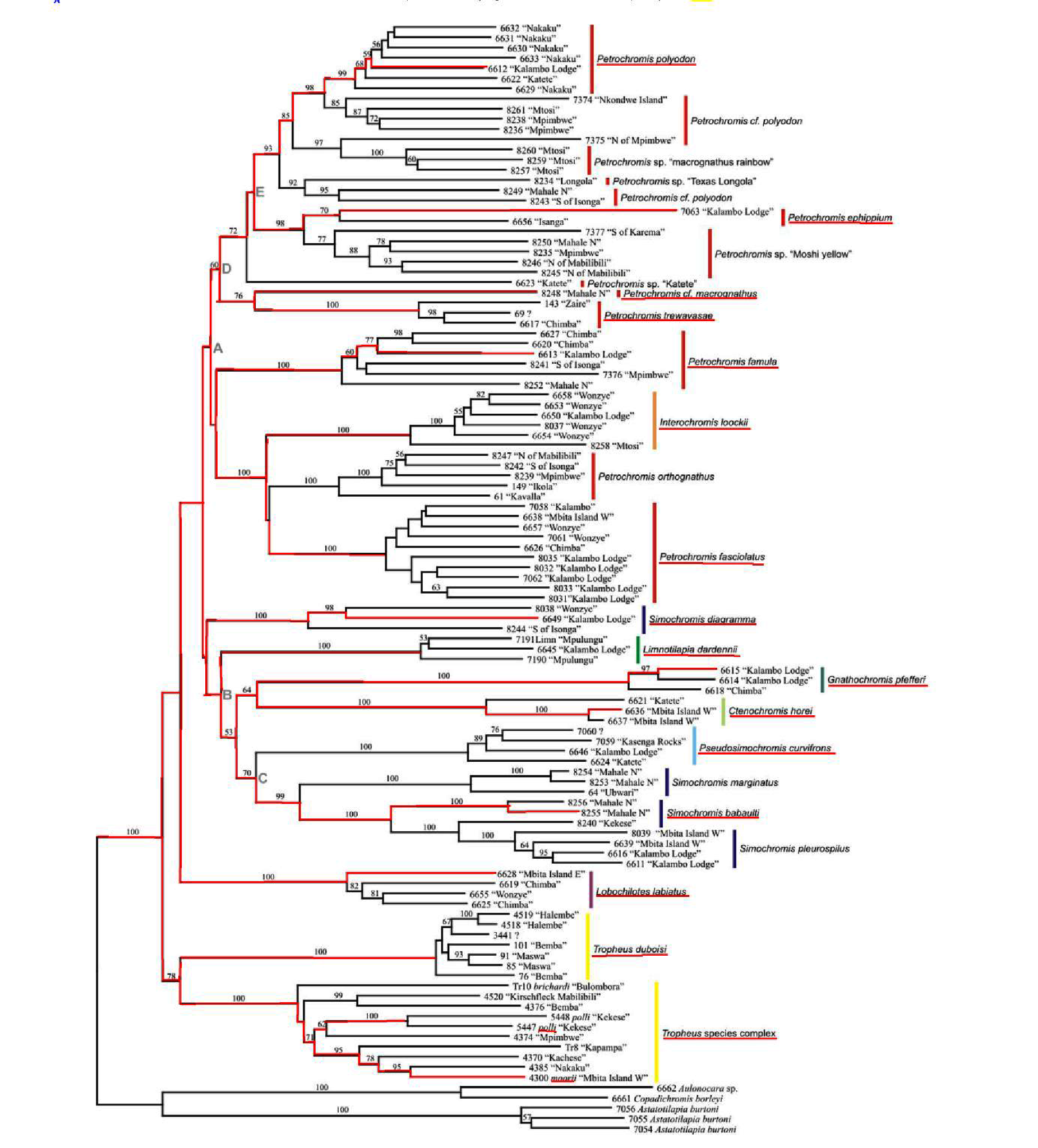

Supplement: Supplementary file 1 — Fig. S1. AFLP‐Phylogeny of the Tropheini after Koblmüller et al. 2010, p. 322. [file ZSC-44-362-s001.tif]

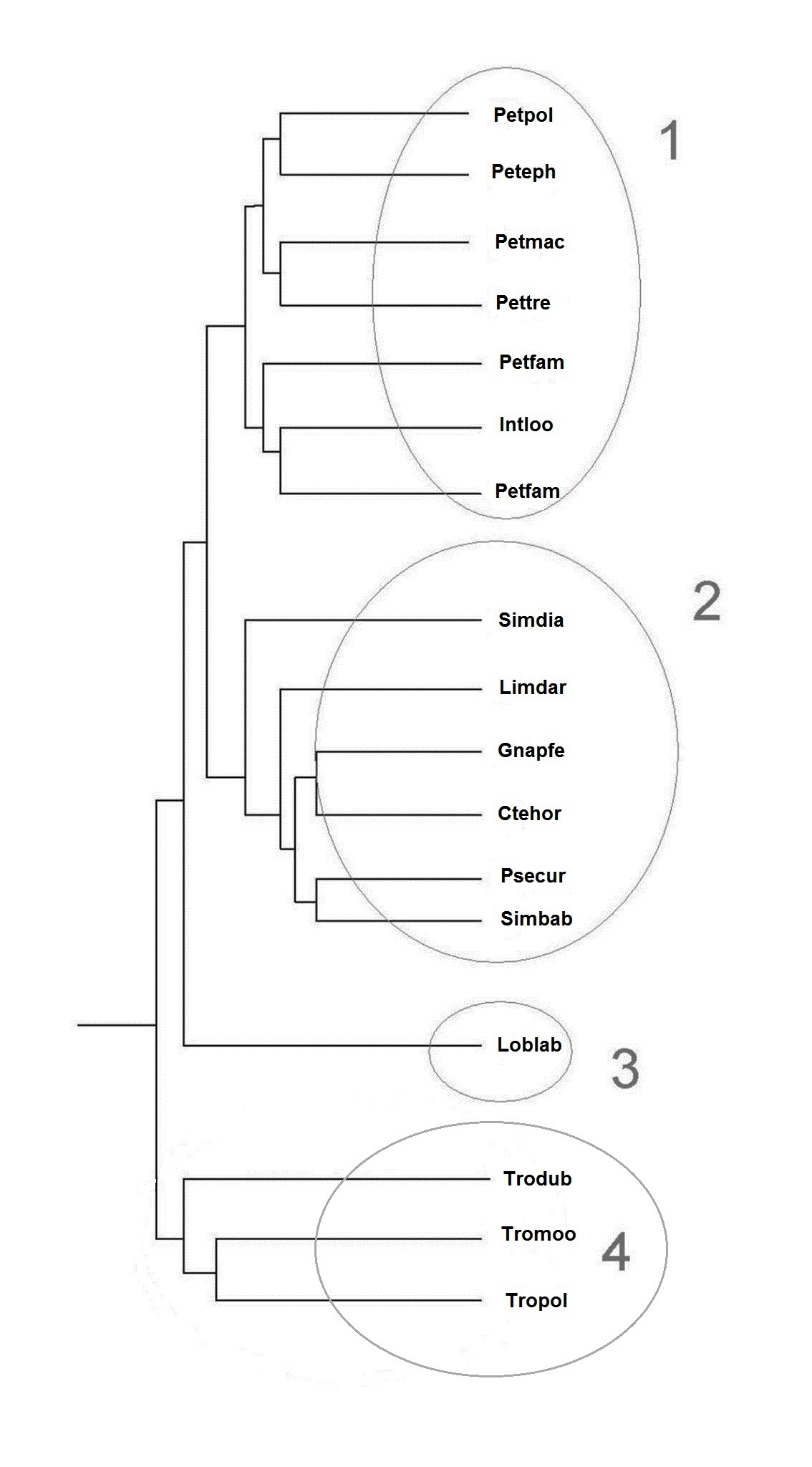

Supplement: Supplementary file 2 — Fig. S2. Phylogeny of the Tropheini, schematically after Koblmüller et al. (2010). [file ZSC-44-362-s002.tif]

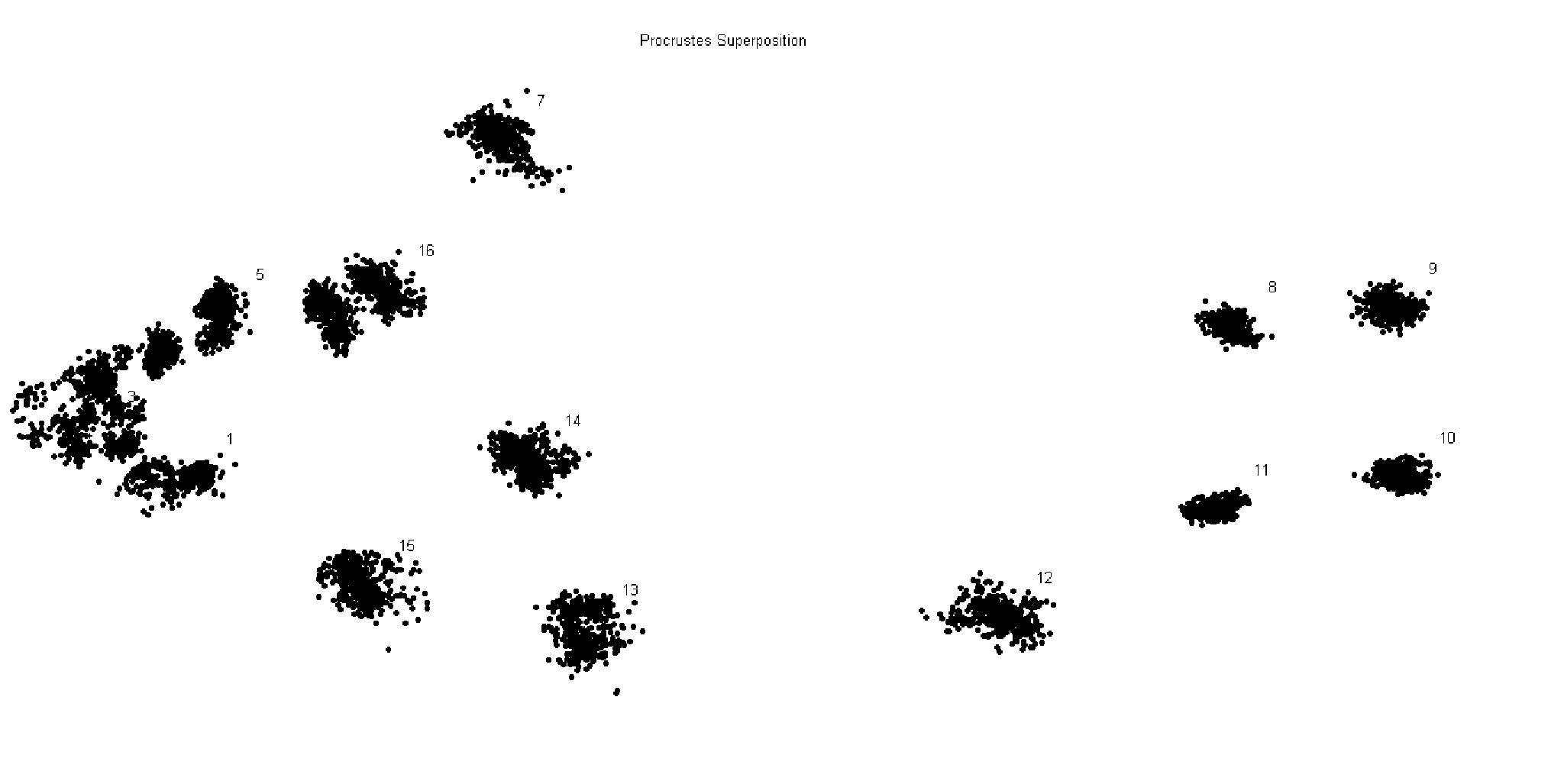

Supplement: Supplementary file 3 — Fig. S3. Procrustes coordinates of all sampled specimens. [file ZSC-44-362-s003.tif]

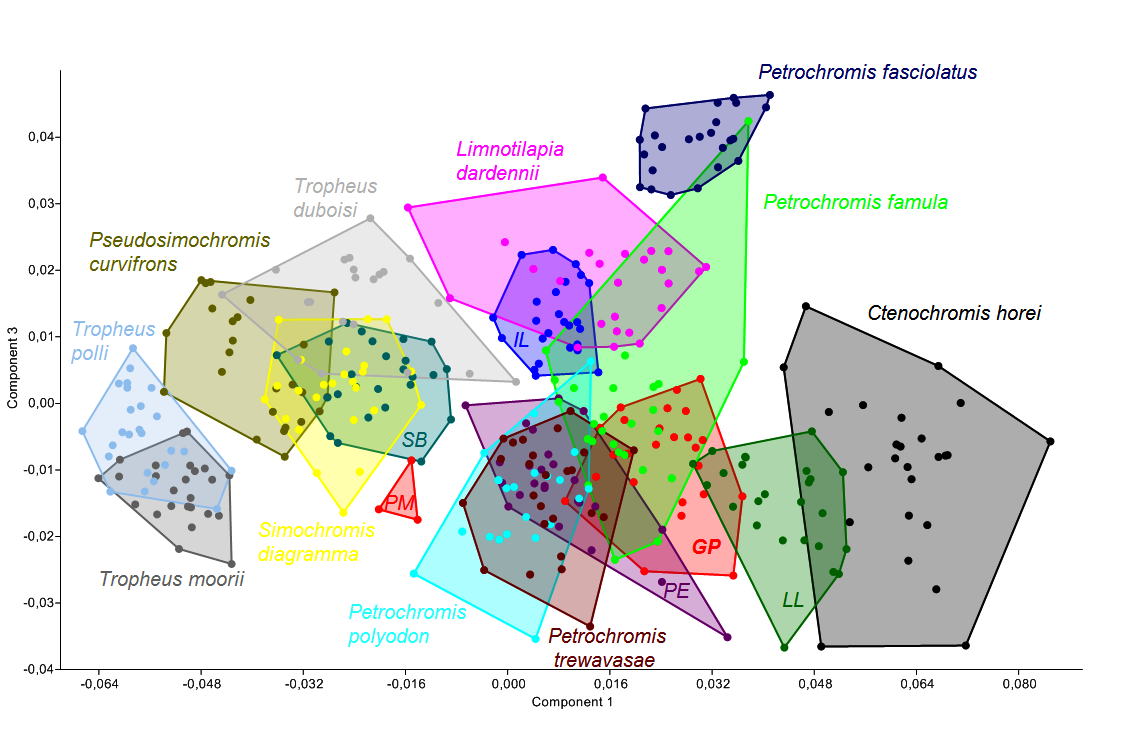

Supplement: Supplementary file 4 — Fig. S4. A: PC1 of all species: PC 1 plotted against PC3. [file ZSC-44-362-s004.tif]

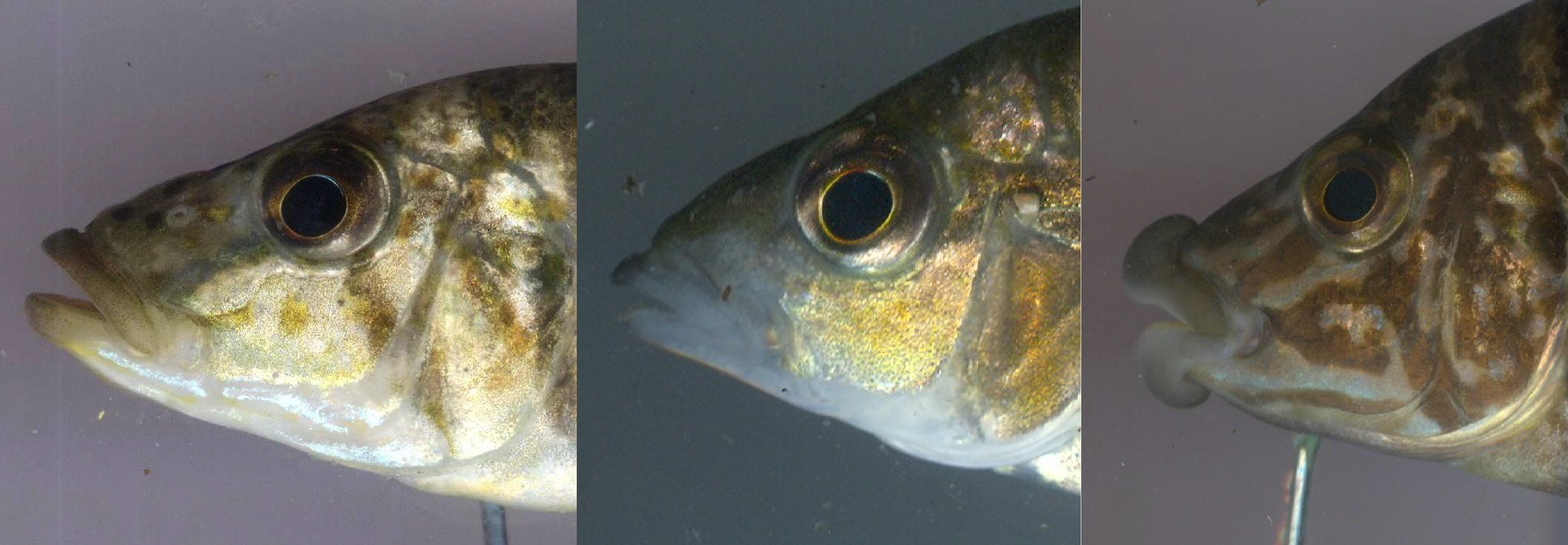

Supplement: Supplementary file 5 — Fig. S5. Heads of Ctenochromis horei (left), Gnathochromis pfefferi (center) and Lobochilotes labiatus (right). [file ZSC-44-362-s005.tif]
