# Supplementary material for: Form, function and phylogeny: comparative morphometrics of Lake Tanganyika's cichlid tribe Tropheini
Source: Zool Scr. 2015 Mar 10;44(4):362–73. doi: 10.1111/zsc.12110 (PMC4949720; doi:10.1111/zsc.12110)
Supplement: Supplementary file 6 — Table S1. Arithmetic mean of standard lengths, standard deviation and coefficient of variation for all examined species. [file ZSC-44-362-s006.docx]

Table S1: Arithmetic mean of standard lengths, standard deviation and coefficient of variation for all examined species

| species | total number | arithmetic mean of standard length[mm] | standard deviation [mm] | coefficient of variation [%] |
| --- | --- | --- | --- | --- |
| *Ctenochromis horei* | 25 | 84.46 | 16.29 | 19.3 |
| *Gnathochromis pfefferi* | 25 | 72.17 | 8.95 | 12.4 |
| *Interochromis loocki* | 25 | 83.98 | 9.69 | 11.5 |
| *Limnotilapia dardennii* | 25 | 81.47 | 30.41 | 37.3 |
| *Lobobchilotes labiatus* | 25 | 90.62 | 13.75 | 15.2 |
| *Petrochromis ephippium* | 25 | 88.51 | 12.84 | 14.5 |
| *Petrochromis famula* | 25 | 71.21 | 14.21 | 19.9 |
| *Petrochromis fasciolatum* | 25 | 77.17 | 6.66 | 8.6 |
| *Petrochromis macrognathus* | 3 | 149.73 | 23.89 | 15.9 |
| *Petrochromis polyodon* | 22 | 128.49 | 21.34 | 16.6 |
| *Petrochromis trewavasae* | 25 | 78.09 | 5.59 | 7.2 |
| *Pseudosimochromis curvifrons* | 25 | 90.94 | 14.9 | 16.4 |
| *Simochromis babaulti* | 25 | 73.18 | 2.79 | 3.8 |
| *Simochromis diagramma* | 25 | 97.16 | 8.33 | 8.6 |
| *Tropheus duboisi* | 21 | 71.43 | 4.82 | 6.8 |
| *Tropheus moorii* | 25 | 76.6 | 2.5 | 3.3 |
| *Tropheus polli* | 25 | 81.62 | 3.5 | 4.3 |
