# Supplementary material for: Form, function and phylogeny: comparative morphometrics of Lake Tanganyika's cichlid tribe Tropheini
Source: Zool Scr. 2015 Mar 10;44(4):362–73. doi: 10.1111/zsc.12110 (PMC4949720; doi:10.1111/zsc.12110)
Supplement: Supplementary file 7 — Table S2. Results from CVA/MANOVA. [file ZSC-44-362-s007.docx]

Table T2: Results from CVA/MANOVA

| Axis | Lambda | Chi square | Degrees of freedom | p |
| --- | --- | --- | --- | --- |
| grouping according to species assignment | | | | |
| 1 | 0.0000 | 6846.87 | 448 | p<2.22045e-016 |
| 2 | 0.0000 | 5488.51 | 405 | p<2.22045e-016 |
| 3 | 0.0000 | 4464.92 | 364 | p<2.22045e-016 |
| 4 | 0.0001 | 3538.78 | 325 | p<2.22045e-016 |
| 5 | 0.0004 | 2925.66 | 288 | p<2.22045e-016 |
| 6 | 0.0017 | 2370.24 | 253 | p<2.22045e-016 |
| 7 | 0.0063 | 1884.82 | 220 | p<2.22045e-016 |
| 8 | 0.0184 | 1488.1 | 189 | p<2.22045e-016 |
| 9 | 0.0486 | 1126.87 | 160 | p<2.22045e-016 |
| 10 | 0.1062 | 835.18 | 133 | p<2.22045e-016 |
| 11 | 0.2101 | 581.17 | 108 | p<2.22045e-016 |
| 12 | 0.3977 | 343.43 | 85 | p<2.22045e-016 |
| 13 | 0.5759 | 205.58 | 64 | p<2.22045e-016 |
| 14 | 0.7306 | 116.93 | 45 | p=2.58764e-008 |
| 15 | 0.8576 | 57.21 | 28 | p=0.000914171 |
| grouping according to trophic guilds | | | | |
| 1 | 0.0055 | 1973.93 | 84 | p<2.22045e-016 |
| 2 | 0.0982 | 879.6 | 54 | p<2.22045e-016 |
| 3 | 0.3743 | 372.45 | 25 | p<2.22045e-016 |
| grouping according to lineages | | | | |
| 1 | 0.0129 | 1647.7 | 84 | p<2.22045e-016 |
| 2 | 0.0829 | 943.75 | 54 | p<2.22045e-016 |
| 3 | 0.3294 | 420.82 | 26 | p<2.22045e-016 |
